# Supplementary material for: MYH3‐associated distal arthrogryposis zebrafish model is normalized with para‐aminoblebbistatin
Source: EMBO Mol Med. 2020 Oct 5;12(11):e12356. doi: 10.15252/emmm.202012356 (PMC7645368; doi:10.15252/emmm.202012356)
Supplement: Supplementary file 1 — Expanded View Figures PDF [file EMMM-12-e12356-s001.pdf]

Expanded View Figures

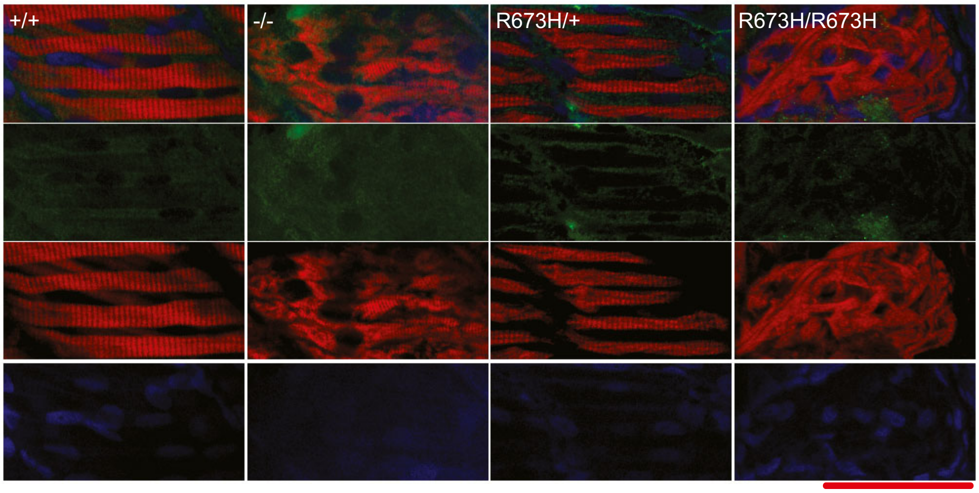

Figure EV1. Transient expression of *smyhc1*.

Smyhc1 immunohistochemistry stain of 3 dpf zebrafish larvae. Filamentous actin is stained with phalloidin (red). Nuclei are stained with DAPI (blue). Smyhc1 is stained with the F59 antibody (green) (Elworthy *et al*, 2008). *smyhc1*$^{+/+}$, *smyhc1*$^{R673H/+}$, and *smyhc1*$^{R673H/R673H}$ larvae do not display Smyhc1 in the muscle fibers at 3dpf.
Data Information: Scale bar represents a length of 50 µm.

© 2020 The Authors

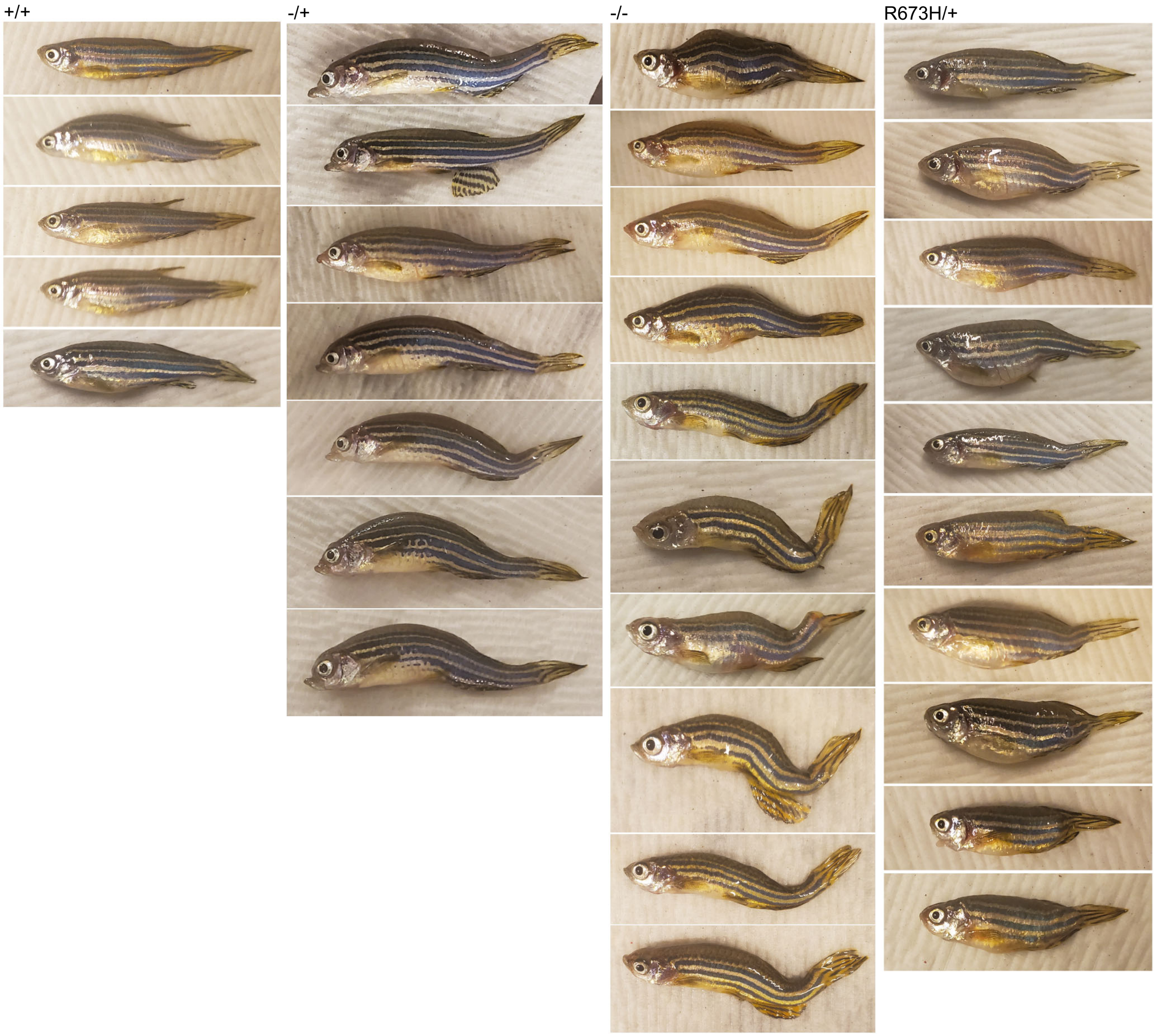

Figure EV2. Examples of adult phenotypes resulting from various *smyhc1* mutant genotypes.

 © 2020 The Authors

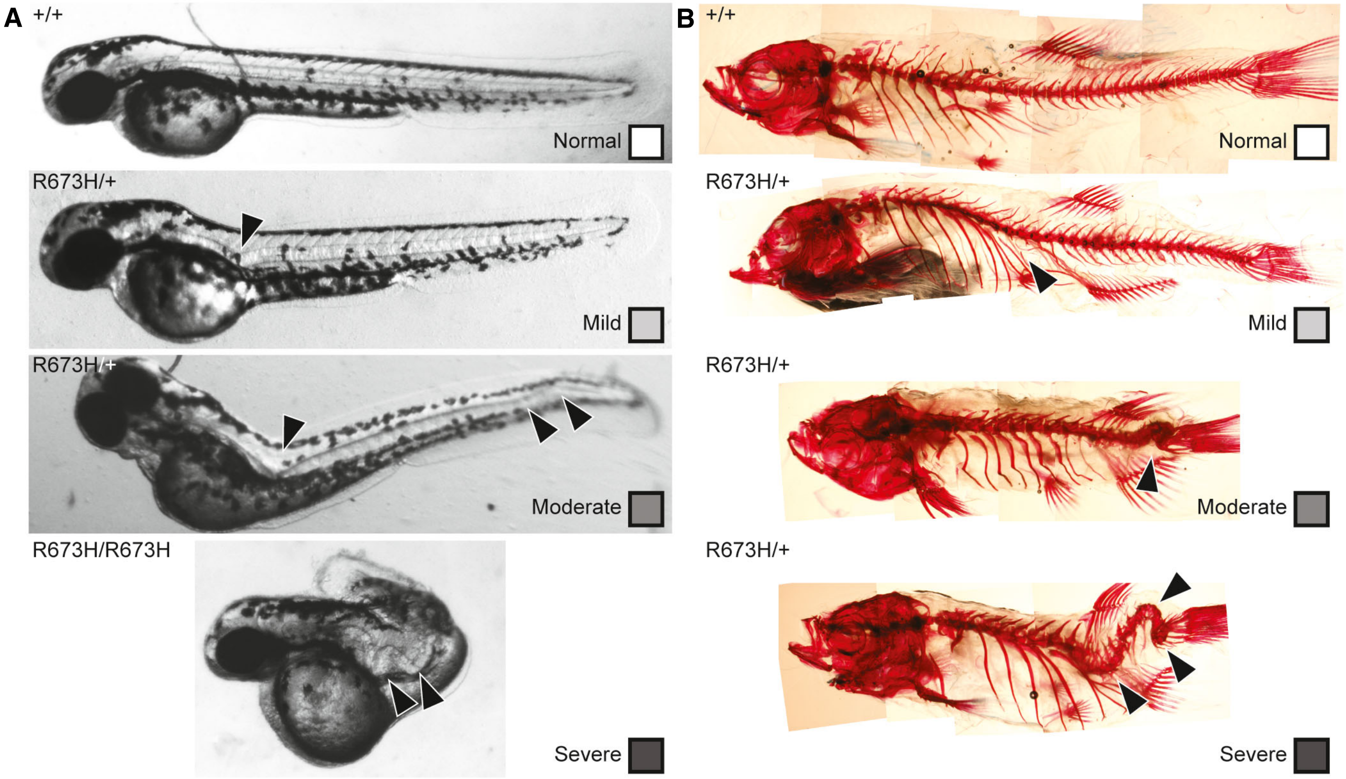

Figure EV3. Representative examples of each embryonic and adult graded phenotype.

A Grading scale for 2 dpf embryos. Normal is classified as 0 visible abnormalities, mild is classified as 1 notochord curve or kink, moderate is classified as multiple notochord curves or kinks, and severe is classified as multiple notochord kinks that severely distort the body. Arrowheads indicate notochord abnormalities.

B Grading scale for adult skeletal abnormalities. Normal is classified as 0 visible abnormalities, mild is classified as 1 spinal curve or kink, moderate is classified as multiple spinal curves or kinks, or apparent vertebral fusion, and severe is classified as multiple spinal kinks or vertebral fusions that severely distort the body. Arrowheads indicate spinal abnormalities.

Figure EV4. Activated Caspase-3 presence in *smyhc1* mutant tissue.

A Confocal fluorescence images of slow skeletal muscle of 1 dpf larvae, stained with phalloidin-rhodamine (red), Caspase-3 antibodies (green), and DAPI (blue).

B Confocal fluorescence images of slow skeletal muscle of 3 dpf larvae, stained with phalloidin-rhodamine (red), Caspase-3 antibodies (green), and DAPI (blue).

Data Information: Scale bars represent a length of 50 µm.

© 2020 The Authors

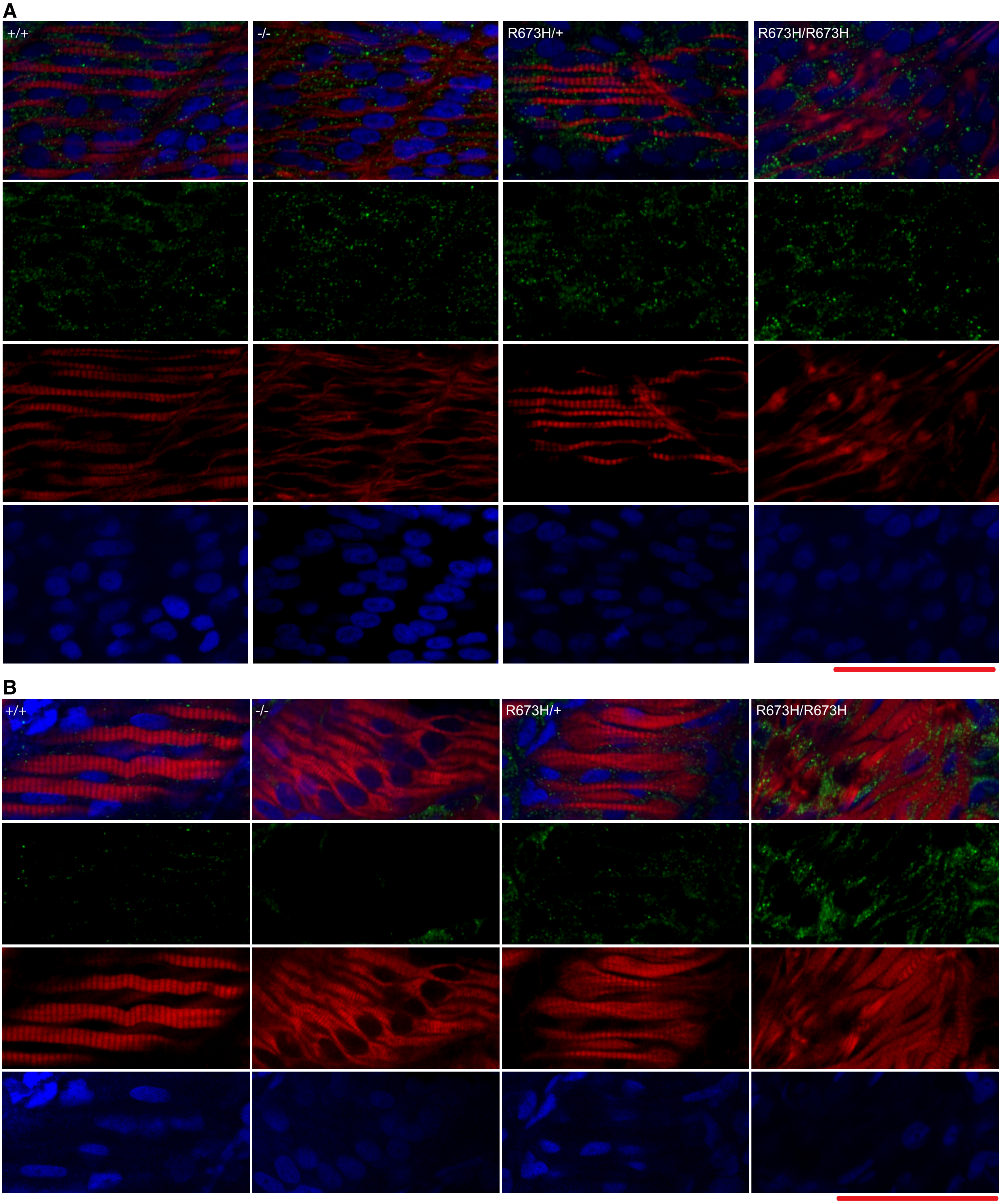

Figure EV4.

© 2020 The Authors

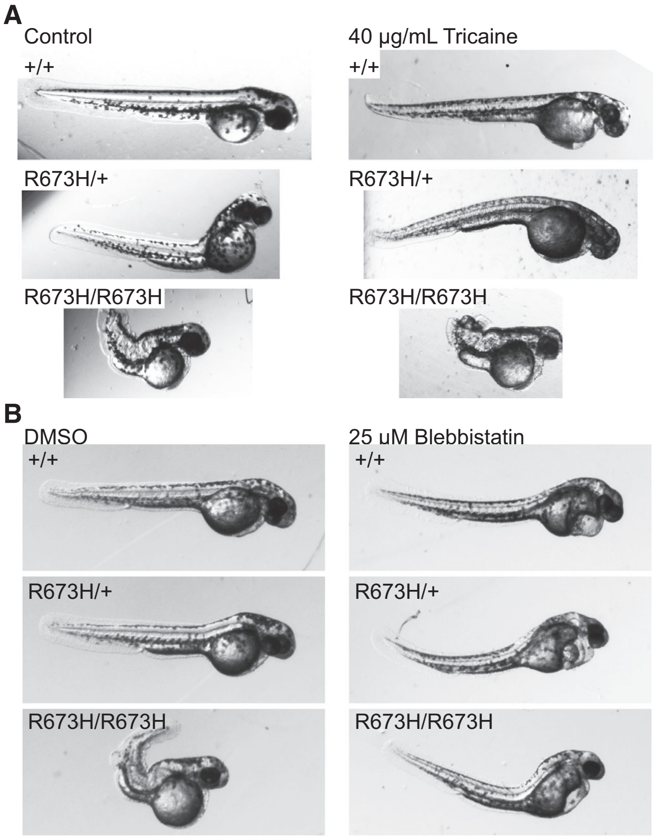

Figure EV5. Tricaine fails to ameliorate the *smyhc1*^{R673H} larval phenotype, and blebbistatin improves the *smyhc1*^{R673H} larval phenotype.

A Zebrafish embryos (2 dpf) treated with 40 mg/ml tricaine methanylsulfonate or control egg medium for 24 h starting at 1 dpf. Tricaine suppressed movement but failed to normalize notochord kinks and bends. Tricaine appears to have no major effect on anatomy.

B Zebrafish embryos (2 dpf) were treated with 25 µM blebbistatin or DMSO control for 24 h starting at 1 dpf. Blebbistatin appears to partially rescue *smyhc1*^{R673H} phenotype, but causes known off target effects. Note the severe dorsal tail curve and pericardial edema present in all larvae treated with blebbistatin regardless of genotype. Representative images of embryos at 48 hpf.
